# Proceedings of the 15th Annual UT-KBRIN Bioinformatics Summit 2016: Cadiz, KY, USA. 8-10 April 2016

**DOI:** 10.1186/s12859-016-1154-y

**Published:** 2016-08-19

**Authors:** Eric C. Rouchka, Julia H. Chariker, Benjamin J. Harrison, Juw Won Park, Xueyuan Cao, Stanley Pounds, Susana Raimondi, James Downing, Raul Ribeiro, Jeffery Rubnitz, Jatinder Lamba, Bernie J. Daigle, Deborah Burgess, Stephanie Gehrlich, John C. Carmen, Nicholas Johnson, Chandrakanth Emani, Stephanie Gehrlich, Deborah Burgess, John C. Carmen, Kalpani De Silva, Michael P. Heaton, Theodore S. Kalbfleisch, Teeradache Viangteeravat, Rahul Mudunuri, Oluwaseun Ajayi, Fatih Şen, Eunice Y. Huang, Mohammad Mohebbi, Luaire Florian, Douglas J. Jackson, John F. Naber, AKM Sabbir, Sally R. Ellingson, Yuping Lu, Charles A Phillips, Michael A. Langston, Rahul K. Sevakula, Raghuveer Thirukovalluru, Nishchal K. Verma, Yan Cui, Mohammed Sayed, Juw Won Park, Jing Wang, Qi Liu, Yu Shyr, Xiaofei Zhang, Sally R. Ellingson, Naresh Prodduturi, Gavin R. Oliver, Diane Grill, Jie Na, Jeanette Eckel-Passow, Eric W. Klee, Michael M. Goodin, Mark Farman, Harrison Inocencio, Chanyong Jang, Jerzy W. Jaromczyk, Neil Moore, Kelly Sovacool, Leon Dent, Mike Izban, Sammed Mandape, Shruti Sakhare, Siddharth Pratap, Dana Marshall, M Scotty DePriest, James N. MacLeod, Theodore S. Kalbfleisch, Chandrakanth Emani, Hanady Adam, Ethan Blandford, Joel Campbell, Joshua Castlen, Brittany Dixon, Ginger Gilbert, Aaron Hall, Philip Kreisle, Jessica Lasher, Bethany Oakes, Allison Speer, Maximilian Valentine, Naga Satya V. Rao Nagisetty, Rony Jose, Teeradache Viangteeravat, Robert Rooney, David Hains

**Affiliations:** 1 Department of Computer Engineering and Computer Science, University of Louisville, Duthie Center for Engineering, Louisville, KY 40292 USA; 2 Kentucky Biomedical Research Infrastructure (KBRIN) Bioinformatics Core, 522 East Gray Street, Louisville, KY 40292 USA; 3 Department of Psychological and Brain Sciences, University of Louisville, Louisville, KY 40292 USA; 4 Department of Anatomical Sciences and Neurobiology, University of Louisville, Louisville, KY 40292 USA; 5 Department of Biostatistics, St. Jude Children’s Research Hospital, Memphis, TN 38105 USA; 6 Department of Pathology, St. Jude Children’s Research Hospital, Memphis, TN 38105 USA; 7 Department of Oncology, St. Jude Children’s Research Hospital, Memphis, TN 38105 USA; 8 Department of Pharmacotherapy and Translational Research, College of Pharmacy, University of Florida, Gainesville, FL 32610 USA; 9 Department of Biological Sciences, University of Memphis, Memphis, TN 38152 USA; 10 Department of Computer Science, University of Memphis, Memphis, TN 38152 USA; 11 Department of Computer Science, Northern Kentucky University, Highland Heights, KY 41099 USA; 12 Department of Biological Sciences, Northern Kentucky University, Highland Heights, KY 41099 USA; 13 Department of Biology, Western Kentucky University, Bowling Green, KY 42101 USA; 14 Department of Biological Sciences, Northern Kentucky University, Highland Heights, KY 41099 USA; 15 Department of Computer Science, Northern Kentucky University, Highland Heights, KY 41099 USA; 16 Interdisciplinary Studies Program: Specialization in Bioinformatics, University of Louisville, Louisville, KY 40292 USA; 17 United States Department of Agriculture, Agricultural Research Service, United States Meat Animal Research Center, Clay Center, NE 68933 USA; 18 Department of Biochemistry and Molecular Genetics, School of Medicine, University of Louisville, Louisville, KY 40292 USA; 19 Biomedical Informatics Core, Children’s Foundation Research Institute, Memphis, TN 38103 USA; 20 Department of Pediatrics, University of Tennessee Health Science Center, Memphis, TN 38163 USA; 21 Department of Electrical and Computer Engineering, University of Louisville, Louisville, KY 40292 USA; 22 Department of Computer Science, University of Kentucky, Lexington, KY 40506 USA; 23 Division of Biomedical Informatics, College of Medicine, University of Kentucky, Lexington, KY 40536-0093 USA; 24 Department of Electrical Engineering and Computer Science, University of Tennessee, Knoxville, TN 37996-2250 USA; 25 Department of Electrical Engineering, Indian Institute of Technology Kanpur, Kanpur, 208016 India; 26 Department of Microbiology, Immunology and Biochemistry, University of Tennessee Health Science Center, Memphis, TN 38163 USA; 27 Department of Computer Engineering and Computer Science, University of Louisville, Louisville, KY 40202 USA; 28 Kentucky Biomedical Research Infrastructure Network (KBRIN) Bioinformatics Core, University of Louisville, Louisville, KY 40292 USA; 29 Center for Quantitative Sciences, Vanderbilt University School of Medicine, Nashville, TN 37232 USA; 30 Department of Cancer Biology, Vanderbilt University School of Medicine, Nashville, TN 37232 USA; 31 Department of Biomedical Informatics, Vanderbilt University School of Medicine, Nashville, TN 37232 USA; 32 Department of Biostatistics, Vanderbilt University School of Medicine, Nashville, TN 37232 USA; 33 Department of Computer Science, University of Kentucky, Lexington, KY 40506 USA; 34 Division of Biomedical Informatics, College of Medicine, University of Kentucky, Lexington, KY 40536-0093 USA; 35 Department of Biomedical Informatics and Statistics, Health Sciences Research, Mayo Clinic, Rochester, MN 55905 USA; 36 Department of Plant Pathology, University of Kentucky, Lexington, KY 40546 USA; 37 Department of Computer Science, University of Kentucky, Lexington, KY 40506 USA; 38 Department of Biology, University of Kentucky, Lexington, KY 40506 USA; 39 Department of Surgery, Meharry Medical College, Nashville, TN 37208 USA; 40 Department of Pathology, Anatomy and Cell Biology, Meharry Medical College, Nashville, TN 37208 USA; 41 Bioinformatics and Proteomics Core, Microbiology and Immunology, Meharry Medical College, Nashville, TN 37208 USA; 42 Department of Biochemistry and Molecular Genetics, School of Medicine, University of Louisville, Louisville, KY 40292 USA; 43 Maxwell H. Gluck Equine Research Center, Department of Veterinary Science, University of Kentucky, Lexington, KY 40546 USA; 44 Department of Biology, Western Kentucky University-Owensboro, Owensboro, KY 42303 USA; 45 Department of Pediatrics, The University of Tennessee Health Science Center, Memphis, TN 38103 USA

## Abstract

I1 Proceedings of the Fifteenth Annual UT- KBRIN Bioinformatics Summit 2016

Eric C. Rouchka, Julia H. Chariker, Benjamin J. Harrison, Juw Won Park

P1 CC-PROMISE: Projection onto the Most Interesting Statistical Evidence (PROMISE) with Canonical Correlation to integrate gene expression and methylation data with multiple pharmacologic and clinical endpoints

Xueyuan Cao, Stanley Pounds, Susana Raimondi, James Downing, Raul Ribeiro, Jeffery Rubnitz, Jatinder Lamba

P2 Integration of microRNA-mRNA interaction networks with gene expression data to increase experimental power

Bernie J Daigle, Jr.

P3 Designing and writing software for *in silico* subtractive hybridization of large eukaryotic genomes

Deborah Burgess, Stephanie Gehrlich, John C Carmen

P4 Tracking the molecular evolution of Pax gene

Nicholas Johnson; Chandrakanth Emani

P5 Identifying genetic differences in thermally dimorphic and state specific fungi using *in silico* genomic comparison

Stephanie Gehrlich, Deborah Burgess, John C Carmen

P6 Identification of conserved genomic regions and variation therein amongst *Cetartiodactyla* species using next generation sequencing

Kalpani De Silva, Michael P Heaton, Theodore S Kalbfleisch

P7 Mining physiological data to identify patients with similar medical events and phenotypes

Teeradache Viangteeravat, Rahul Mudunuri, Oluwaseun Ajayi, Fatih Şen, Eunice Y Huang

P8 Smart brief for home health monitoring

Mohammad Mohebbi, Luaire Florian, Douglas J Jackson, John F Naber

P9 Side-effect term matching for computational adverse drug reaction predictions

AKM Sabbir, Sally R Ellingson

P10 Enrichment vs robustness: A comparison of transcriptomic data clustering metrics

Yuping Lu, Charles A Phillips, Michael A Langston

P11 Deep neural networks for transcriptome-based cancer classification

Rahul K Sevakula, Raghuveer Thirukovalluru, Nishchal K. Verma, Yan Cui

P12 Motif discovery using K-means clustering

Mohammed Sayed, Juw Won Park

P13 Large scale discovery of active enhancers from nascent RNA sequencing

Jing Wang, Qi Liu, Yu Shyr

P14 Computationally characterizing genomic pipelines and benchmarking results using GATK best practices on the high performance computing cluster at the University of Kentucky

Xiaofei Zhang, Sally R Ellingson

P15 Development of approaches enabling the identification of abnormal gene expression from RNA-Seq in personalized oncology

Naresh Prodduturi, Gavin R Oliver, Diane Grill, Jie Na, Jeanette Eckel-Passow, Eric W Klee

P16 Processing RNA-Seq data of plants infected with coffee ringspot virus

Michael M Goodin, Mark Farman, Harrison Inocencio, Chanyong Jang, Jerzy W Jaromczyk, Neil Moore, Kelly Sovacool

P17 Comparative transcriptomics of three *Acinetobacter baumanii* clinical isolates with different antibiotic resistance patterns

Leon Dent, Mike Izban, Sammed Mandape, Shruti Sakhare, Siddharth Pratap, Dana Marshall

P18 Metagenomic assessment of possible microbial contamination in the equine reference genome assembly

M Scotty DePriest, James N MacLeod, Theodore S Kalbfleisch

P19 Molecular evolution of cancer driver genes

Chandrakanth Emani, Hanady Adam, Ethan Blandford, Joel Campbell, Joshua Castlen, Brittany Dixon, Ginger Gilbert, Aaron Hall, Philip Kreisle, Jessica Lasher, Bethany Oakes, Allison Speer, Maximilian Valentine

P20 Biorepository Laboratory Information Management System

Naga Satya V Rao Nagisetty, Rony Jose, Teeradache Viangteeravat, Robert Rooney, David Hains

## I1 Proceedings of the Fifteenth Annual UT- KBRIN Bioinformatics Summit 2016

### Eric C. Rouchka^1,2^, Julia H. Chariker^2,3^ (julia.chariker@louisville.edu), Benjamin J. Harrison^2,4^ (b.harrison@louisville.edu), Juw Won Park^1,2^ (juw.park@louisville.edu)

#### ^1^Department of Computer Engineering and Computer Science, University of Louisville, Duthie Center for Engineering, Louisville, KY 40292, USA; ^2^Kentucky Biomedical Research Infrastructure (KBRIN) Bioinformatics Core, 522 East Gray Street, Louisville, KY 40292, USA; ^3^Department of Psychological and Brain Sciences, University of Louisville, Louisville, KY 40292, USA; ^4^Department of Anatomical Sciences and Neurobiology, University of Louisville, Louisville, KY 40292, USA

##### **Correspondence:** Eric C. Rouchka (eric.rouchka@louisville.edu) – Kentucky Biomedical Research Infrastructure (KBRIN) Bioinformatics Core, 522 East Gray Street, Louisville, KY 40292, USA

The University of Tennessee (UT) and the Kentucky Biomedical Research Infrastructure Network (KBRIN) have collaborated over the past fifteen years to share research and educational expertise in bioinformatics. One result is an annual regional summit for researchers, educators and students. The Fifteenth Annual UT-KBRIN Bioinformatics Summit was held at Lake Barkley State Park from April 8-10, 2016. A total of 184 participants pre-registered, with 85 from Tennessee, 84 from Kentucky, and the remainder from various states and international locales. Among the registrants were 64 faculty, 47 staff, 46 students, and 20 postdocs. The conference program consisted of two workshops on the UCSC Genome Browser and two days of plenary presentations and short talks. In addition, a poster session with 57 posters was held on Saturday evening.

**Friday Workshops**

Robert Kuhn (University of California – Santa Cruz) opened the Summit with the workshop “UCSC Genome Browser Part I: Visualization Tool for Genomes.” This workshop focused on a lecture and live demonstration of the capabilities of the UCSC Genome Browser (http://genome.ucsc.edu/) [1-3]. Dr. Kuhn talked about many of the capabilities of the browser, including the use of pre-defined annotation tracks, the creation and use of user-defined annotation tracks and track hubs [4], connecting to the annotation tables via database connections [5] and the ability to implement protected sessions through the Genome Browser in a Box [6]. After a short break, Dr. Kuhn continued with “UCSC Genome Browser Part II: Visualization tool for genomes hands-on experience.” In this workshop, Dr. Kuhn focused on use of the UCSC Genome Browser through a set of user-guided exercises intended to give participants hands-on experience with both basic and advanced features of the genome browser.

**Session I**

GQ Zhang (University of Kentucky) opened the scientific session on Saturday morning with a presentation on “The role of ontologies in clinical and translational informatics.” His talk was broken down into three areas based on biomedical ontologies, ontologies in use, and ontology quality assurance. In the first portion, Dr. Zhang discussed biomedical ontologies in general, particularly in terms of the resources available to the University of Kentucky Center for Clinical and Translational Science, including the Appalachian Translational Research Network which consists of clinical and translational partners across Kentucky, Tennessee, Ohio, and West Virginia. Dr. Zhang discussed some of the challenges in scaling up translational informatics using big data from a patient perspective to create a learning health care system. In the second portion, Dr. Zhang discussed two resources, including the National Sleep Research Resource (sleepdata.org) [7] and the Center for Sudep Research [8-10]. In the final portion, Dr. Zhang discussed ontology quality assurance with a specific example using Gene Ontology [11] fragments and SNOMED [12, 13] data and approaches his group has taken towards developing algorithms for ontology quality assurance [14-16].

Igor Jouline (Oak Ridge National Laboratory and The University of Tennessee – Knoxville) followed with the plenary talk “Using evolutionary history for predicting functional changes in proteins.” This presentation focused on the use of evolutionary and conserved core elements within systems, such as signal transduction and chemotaxis systems within bacteria, in order to predict likely functional changes [17-21]. Dr. Jouline gave additional examples, including the structural diversity of chemoreceptor signaling domains [22-24]. Dr. Jouline brought home the point of how critical it is to consider the phylogenetic history from a sequence point of view in order to help classify disease mutations [25]. He introduced a computational approach his group has developed which looks at evolutionary changes in genes and their paralogs, and showed that changes need to be considered across all copies, in order to fully understand disease implications [26].

**Session II**

Nancy Cox (Vanderbilt University) led the second session on Saturday morning with a presentation titled “Building a catalog of gene to medical phenome: New ways of understanding the biological mechanisms of disease.” In this presentation, Dr. Cox presented PrediXcan, an approach to medical informatics data integration [27]. Highlighted within this talk were preliminary results of applying PredicXcan to Vanderbilt University’s BioVU [28] which contains several over 215,000 subjects with DNA. Within this dataset are approximately 20,000 dense GWAS genotypes and 42,000 exome chips. Dr. Cox discussed how nearly all genes have a high correlation in at least one tissue type, with 4,000-9,000 correlating within any given tissue. Among the research results presented were several novel gene-phenotype relationships built upon disease models of genetically regulated expression (GReX) [27] and genotype tissue expression (GTEx) [29]. Dr. Cox discussed how disease from a gene expression point of view can be explained by major axes of disease risk, in which the healthiest individuals maintain a balance in the center of all of the axes.

**Session III**

Ting Wang (Washington University in St. Louis) opened up the final plenary session with a presentation “Epigenetics roadmap.” Dr. Wang’s talk was broken down into several sections. The first part of his talk focused on discussion of the Roadmap Epigenomics Project [30, 31] which collected a variety of epigenetic markers, including DNA methylation, open chromatin, and histone modification on over 100 tissue and cell types. The second portion of his talk focused on methods of accessing the Roadmap Epigenomics data, including tracks within the UCSC Genome Browser [4] and the WashU Epigenome Browser [32, 33] developed within his group. In the third section, Dr. Wang discussed project extensions, including the recent 4D Nucleosome [34] which focuses on integration of genomic and imaging data and TaRGET project which will focus on epigenomic changes relative to toxicants. The fourth and final portion of the talk was dedicated to discoveries aided by the Roadmap Epigenomics, including epigenetic annotation of genetic variants associated with disease [35] and genetic regulation due to transposable elements [36, 37].

The final plenary speaker on Sunday was Csaba Kovesdy from the University of Tennessee Health Science Center. He presented “Modeling clinical trials using observational methods: How Big Data can help us.” During the course of this presentation, Dr. Kovesdy discussed the use of Big Data in health care from the Veterans Administration (VA) in terms of making discovery in chronic kidney disease (CKD). Dr. Kovesdy proposed how Big Data might be used to supplement the use of clinical trials, particularly in cases when a study of interest is a subset of a larger study. In one particular case, Dr. Kovesdy discussed using data from large studies on hypertension to make discoveries in CKD [38-40]. In addition, he discussed strengths and weaknesses of dealing with data from the VA patient population, and potential future opportunities with the Million Veteran Program [41].

**Poster Session**

A poster session and reception was held on Saturday evening with a total of 57 posters presented across 15 categories. The largest represented categories included high throughput sequencing, bioinformatics of health and disease, systems biology and networks, and comparative genomics. 24 of the poster abstracts are highlighted within this supplement. Eight of the poster abstracts were selected for short 10 minute presentations at the summit, including “Identifying clusters in protein structure: Comparisons between polymorphic, pathogenic, and somatic variation” (R. Michael Sivley, Vanderbilt University); “*Porphyromonas gingivalis* and the epithelial-to-mesenchymal transition (EMT): Signaling networks linking infection to cancer promotion” (Melissa Metzler, University of Louisville); “CC-PROMISE: Projection onto the most interesting statistical evidence (PROMISE) with canonical correlation to integrate gene expression and methylation data with multiple pharmacologic and clinical endpoints” (Xueyuan Cao, St. Jude Children’s Research Hospital); “Biochemically Aware Substructure Search (BASS) – An algorithm for finding biochemically relevant chemical subgraphs” (Joshua Mitchell, University of Kentucky); “Integration of microRNA-mRNA interaction networks with gene expression data to increase experimental power” (Bernie Daigle, University of Memphis); “Side-effect term matching for computational adverse drug predictions” (AKM Sabbir, University of Kentucky); “Lentiviral CRISPR/cas9 vector mediated miRNA editing disrupts miRNA function” (Junming Yue, University of Tennessee Health Science Center); and “CSI-UTR: An algorithm for characterizing 3’ untranslated region (3’ UTR) diversity in RNA-Seq data by employing RNA cleavage site intervals (CSIs)” (Ben Harrison, University of Louisville).

**Future Plans**

The 2017 Summit is scheduled for March 20-22 at Montgomery Bell State Park in Tennessee. Sessions will focus more on bioinformatics research in Kentucky and Tennessee to help forge more collaborative efforts. Plans are to include more Tennessee and Kentucky speakers, in terms of intermediate length (20-30 minute) presentations and short (10 minute) presentations that complement plenary session topics as well as integrating 1 minute flash talk opportunities for those submitting poster abstracts.

**Acknowledgements**

We would like to thank the Conference Program Committee members Hao Chen (University of Tennessee Health Science Center), Nigel Cooper (University of Louisville), Dan Goldowitz (University of British Columbia), Mike Langston (University of Tennessee-Knoxville), Terry Mark-Major (University of Tennessee Health Science Center), Hunter Moseley (University of Kentucky), Juw Won Park (University of Louisville), Claire Rinehart (Western Kentucky University), Arnold Stromberg (University of Kentucky), Rob Williams (University of Tennessee Health Science Center), and Zhongming Zhao (University of Texas Health Science Center - Houston) for organizing an outstanding scientific program. In addition, we wish to thank Susan Boucher, Alicia Brookins, Terry Mark-Major, Michelle Padgett, and Whitney Rogers for their efforts in handling conference organization details. Funding for the UT- KBRIN Summit is provided in part by the University of Memphis Office of the Provost, Memphis Research Consortium, Kentucky Biomedical Research Infrastructure Network (KBRIN), University of Tennessee Center for Integrative and Translational Genomics, University of Tennessee Molecular Resource Center, and NIH grant P20GM103436.

**References**

1. Kent WJ, Sugnet CW, Furey TS, Roskin KM, Pringle TH, Zahler AM, Haussler D: **The human genome browser at UCSC**. *Genome Res* 2002, **12**(6):996-1006.

2. Kuhn RM, Haussler D, Kent WJ: **The UCSC genome browser and associated tools**. *Brief Bioinform* 2013, **14**(2):144-161.

3. Speir ML, Zweig AS, Rosenbloom KR, Raney BJ, Paten B, Nejad P, Lee BT, Learned K, Karolchik D, Hinrichs AS *et al*: **The UCSC Genome Browser database: 2016 update**. *Nucleic Acids Res* 2016, **44**(D1):D717-725.

4. Raney BJ, Dreszer TR, Barber GP, Clawson H, Fujita PA, Wang T, Nguyen N, Paten B, Zweig AS, Karolchik D *et al*: **Track data hubs enable visualization of user-defined genome-wide annotations on the UCSC Genome Browser**. *Bioinformatics* 2014, **30**(7):1003-1005.

5. Karolchik D, Hinrichs AS, Furey TS, Roskin KM, Sugnet CW, Haussler D, Kent WJ: **The UCSC Table Browser data retrieval tool**. *Nucleic Acids Res* 2004, **32**(Database issue):D493-496.

6. Haeussler M, Raney BJ, Hinrichs AS, Clawson H, Zweig AS, Karolchik D, Casper J, Speir ML, Haussler D, Kent WJ: **Navigating protected genomics data with UCSC Genome Browser in a Box**. *Bioinformatics* 2015, **31**(5):764-766.

7. Redline S, Sanders MH, Lind BK, Quan SF, Iber C, Gottlieb DJ, Bonekat WH, Rapoport DM, Smith PL, Kiley JP: **Methods for obtaining and analyzing unattended polysomnography data for a multicenter study. Sleep Heart Health Research Group**. *Sleep* 1998, **21**(7):759-767.

8. Sahoo SS, Lhatoo SD, Gupta DK, Cui L, Zhao M, Jayapandian C, Bozorgi A, Zhang GQ: **Epilepsy and seizure ontology: towards an epilepsy informatics infrastructure for clinical research and patient care**. *J Am Med Inform Assoc* 2014, **21**(1):82-89.

9. Sahoo SS, Zhang GQ, Bamps Y, Fraser R, Stoll S, Lhatoo SD, Tatsuoka C, Sams J, Welter E, Sajatovic M: **Managing information well: Toward an ontology-driven informatics platform for data sharing and secondary use in epilepsy self-management research centers**. *Health Informatics J* 2015.

10. Zhang GQ, Cui L, Lhatoo S, Schuele SU, Sahoo SS: **MEDCIS: Multi-Modality Epilepsy Data Capture and Integration System**. *AMIA Annu Symp Proc* 2014, **2014**:1248-1257.

11. Ashburner M, Ball CA, Blake JA, Botstein D, Butler H, Cherry JM, Davis AP, Dolinski K, Dwight SS, Eppig JT *et al*: **Gene ontology: tool for the unification of biology. The Gene Ontology Consortium**. *Nat Genet* 2000, **25**(1):25-29.

12. Cote RA, Robboy S: **Progress in medical information management. Systematized nomenclature of medicine (SNOMED)**. *JAMA* 1980, **243**(8):756-762.

13. Sun M, Zhu W, Tao S, Cui L, Zhang GQ: **COBE: A Conjunctive Ontology Browser and Explorer for Visualizing SNOMED CT Fragments**. *AMIA Annu Symp Proc* 2015, **2015**:2092-2100.

14. Jayapandian C, Wei A, Ramesh P, Zonjy B, Lhatoo SD, Loparo K, Zhang GQ, Sahoo SS: **A scalable neuroinformatics data flow for electrophysiological signals using MapReduce**. *Front Neuroinform* 2015, **9**:4.

15. Zhang GQ, Bodenreider O: **Using SPARQL to Test for Lattices: application to quality assurance in biomedical ontologies**. *Semant Web ISWC* 2010, **6497**:273-288.

16. Zhang GQ, Zhu W, Sun M, Tao S, Bodenreider O, Cui L: **MaPLE: A MapReduce Pipeline for Lattice-based Evaluation and Its Application to SNOMED CT**. *Proc IEEE Int Conf Big Data* 2014, **2014**:754-759.

17. Cashman DJ, Ortega DR, Zhulin IB, Baudry J: **Homology modeling of the CheW coupling protein of the chemotaxis signaling complex**. *PLoS One* 2013, **8**(8):e70705.

18. Ortega DR, Zhulin IB: **Evolutionary Genomics Suggests That CheV Is an Additional Adaptor for Accommodating Specific Chemoreceptors within the Chemotaxis Signaling Complex**. *PLoS Comput Biol* 2016, **12**(2):e1004723.

19. Wuichet K, Alexander RP, Zhulin IB: **Comparative genomic and protein sequence analyses of a complex system controlling bacterial chemotaxis**. *Methods Enzymol* 2007, **422**:1-31.

20. Wuichet K, Zhulin IB: **Origins and diversification of a complex signal transduction system in prokaryotes**. *Sci Signal* 2010, **3**(128):ra50.

21. Xie Z, Ulrich LE, Zhulin IB, Alexandre G: **PAS domain containing chemoreceptor couples dynamic changes in metabolism with chemotaxis**. *Proc Natl Acad Sci U S A* 2010, **107**(5):2235-2240.

22. Alexander RP, Zhulin IB: **Evolutionary genomics reveals conserved structural determinants of signaling and adaptation in microbial chemoreceptors**. *Proc Natl Acad Sci U S A* 2007, **104**(8):2885-2890.

23. Ortega DR, Yang C, Ames P, Baudry J, Parkinson JS, Zhulin IB: **A phenylalanine rotameric switch for signal-state control in bacterial chemoreceptors**. *Nat Commun* 2013, **4**:2881.

24. Upadhyay AA, Fleetwood AD, Adebali O, Finn RD, Zhulin IB: **Cache Domains That are Homologous to, but Different from PAS Domains Comprise the Largest Superfamily of Extracellular Sensors in Prokaryotes**. *PLoS Comput Biol* 2016, **12**(4):e1004862.

25. Adebali O, Reznik AO, Ory DS, Zhulin IB: **Establishing the precise evolutionary history of a gene improves prediction of disease-causing missense mutations**. *Genet Med* 2016.

26. Adebali O, Ortega DR, Zhulin IB: **CDvist: a webserver for identification and visualization of conserved domains in protein sequences**. *Bioinformatics* 2015, **31**(9):1475-1477.

27. Gamazon ER, Wheeler HE, Shah KP, Mozaffari SV, Aquino-Michaels K, Carroll RJ, Eyler AE, Denny JC, Consortium GT, Nicolae DL *et al*: **A gene-based association method for mapping traits using reference transcriptome data**. *Nat Genet* 2015, **47**(9):1091-1098.

28. Pulley J, Clayton E, Bernard GR, Roden DM, Masys DR: **Principles of human subjects protections applied in an opt-out, de-identified biobank**. *Clin Transl Sci* 2010, **3**(1):42-48.

29. Wang J, Gamazon ER, Pierce BL, Stranger BE, Im HK, Gibbons RD, Cox NJ, Nicolae DL, Chen LS: **Imputing Gene Expression in Uncollected Tissues Within and Beyond GTEx**. *Am J Hum Genet* 2016, **98**(4):697-708.

30. Bernstein BE, Stamatoyannopoulos JA, Costello JF, Ren B, Milosavljevic A, Meissner A, Kellis M, Marra MA, Beaudet AL, Ecker JR *et al*: **The NIH Roadmap Epigenomics Mapping Consortium**. *Nat Biotechnol* 2010, **28**(10):1045-1048.

31. Roadmap Epigenomics C, Kundaje A, Meuleman W, Ernst J, Bilenky M, Yen A, Heravi-Moussavi A, Kheradpour P, Zhang Z, Wang J *et al*: **Integrative analysis of 111 reference human epigenomes**. *Nature* 2015, **518**(7539):317-330.

32. Zhou X, Li D, Lowdon RF, Costello JF, Wang T: **methylC Track: visual integration of single-base resolution DNA methylation data on the WashU EpiGenome Browser**. *Bioinformatics* 2014, **30**(15):2206-2207.

33. Zhou X, Wang T: **Using the Wash U Epigenome Browser to examine genome-wide sequencing data**. *Curr Protoc Bioinformatics* 2012, **Chapter 10**:Unit10 10.

34. Tashiro S, Lanctot C: **The International Nucleome Consortium**. *Nucleus* 2015, **6**(2):89-92.

35. Zhou X, Li D, Zhang B, Lowdon RF, Rockweiler NB, Sears RL, Madden PA, Smirnov I, Costello JF, Wang T: **Epigenomic annotation of genetic variants using the Roadmap Epigenome Browser**. *Nat Biotechnol* 2015, **33**(4):345-346.

36. Sundaram V, Cheng Y, Ma Z, Li D, Xing X, Edge P, Snyder MP, Wang T: **Widespread contribution of transposable elements to the innovation of gene regulatory networks**. *Genome Res* 2014, **24**(12):1963-1976.

37. Xie M, Hong C, Zhang B, Lowdon RF, Xing X, Li D, Zhou X, Lee HJ, Maire CL, Ligon KL *et al*: **DNA hypomethylation within specific transposable element families associates with tissue-specific enhancer landscape**. *Nat Genet* 2013, **45**(7):836-841.

38. Gosmanov AR, Lu JL, Sumida K, Potukuchi PK, Rhee CM, Kalantar-Zadeh K, Molnar MZ, Kovesdy CP: **Synergistic association of combined glycemic and blood pressure level with risk of complications in US veterans with diabetes**. *J Hypertens* 2016, **34**(5):907-913.

39. Kovesdy CP, Bleyer AJ, Molnar MZ, Ma JZ, Sim JJ, Cushman WC, Quarles LD, Kalantar-Zadeh K: **Blood pressure and mortality in U.S. veterans with chronic kidney disease: a cohort study**. *Ann Intern Med* 2013, **159**(4):233-242.

40. Li L, Streja E, Rhee CM, Mehrotra R, Soohoo M, Brunelli SM, Kovesdy CP, Kalantar-Zadeh K: **Hypomagnesemia and Mortality in Incident Hemodialysis Patients**. *Am J Kidney Dis* 2015, **66**(6):1047-1055.

41. Gaziano JM, Concato J, Brophy M, Fiore L, Pyarajan S, Breeling J, Whitbourne S, Deen J, Shannon C, Humphries D *et al*: **Million Veteran Program: A mega-biobank to study genetic influences on health and disease**. *J Clin Epidemiol* 2016, **70**:214-223.

## P1 CC-PROMISE: Projection onto the Most Interesting Statistical Evidence (PROMISE) with Canonical Correlation to integrate gene expression and methylation data with multiple pharmacologic and clinical endpoints

### Xueyuan Cao^1^, Stanley Pounds^1^, Susana Raimondi^2^, James Downing^2^, Raul Ribeiro^3^, Jeffery Rubnitz^3^, Jatinder Lamba^4^

#### ^1^Department of Biostatistics, St. Jude Children's Research Hospital, Memphis, TN 38105, USA; ^2^Department of Pathology, St. Jude Children's Research Hospital, Memphis, TN 38105, USA; ^3^Department of Oncology, St. Jude Children's Research Hospital, Memphis, TN 38105, USA; ^4^Department of Pharmacotherapy and Translational Research, College of Pharmacy, University of Florida, Gainesville, FL 32610, USA

##### **Correspondence:** Stanley Pounds (stanley.pounds@stjude.org) – Department of Biostatistics, St. Jude Children's Research Hospital, Memphis, TN 38105, USA

**Background**

Projection onto the most interesting statistical evidence (PROMISE) is a general procedure to identify genomic variables that exhibit a specific biologically interesting pattern of association with multiple endpoint variables. It has been successfully applied to multiple studies with gene expression profiling or SNP data to identify genes that are associated with intrinsically related clinical endpoint variables in cancer. In the context of genetic studies in clinical trials, the clinical endpoint variables are related to one another and different forms of genetic data (such as methylation and expression) are also related to one another.

**Materials and methods**

Here, CC-PROMISE, PROMISE with canonical correlation, is proposed to extend the PROMISE procedure to integrate DNA methylation, gene expression and multiple pharmacologic and/or clinical variables into one test at the gene level. First, canonical correlation analysis is performed on the multiple probe-sets measuring DNA methylation and gene expression in one gene. Second, the methylation and expression scores of the gene are calculated as the first canonical correlates of the signals of individual methylation probes and expression probes, respectively. Third, perform PROMISE analysis with multiple endpoints on the methylation scores and expression scores separately. Next, the CC-PROMISE statistic is defined as the average of the PROMISE statistics of the methylation and expression scores. Finally, the significance of CC-PROMISE statistics is determined by permuting the endpoint data.

**Results**

We applied the CC-PROMISE procedure to the Affymetrix U133A gene expression array and Illumina 450K methylation array data of the multi-center AML02 clinical trial (NCT00136084). The methylation and expression of 202 genes showed a meaningful pattern of association with in vitro drug sensitivity, minimal residual disease, and event-free survival (p ≤ 0.001, q ≤ 0.05). Several of the identified genes are of known relevance to disease biology, showing that CC-PROMISE can make meaningful discoveries by effectively integrating expression, methylation, and clinical data.

## P2 Integration of microRNA-mRNA interaction networks with gene expression data to increase experimental power

### Bernie J Daigle, Jr.^1,2^ (bjdaigle@memphis.edu)

#### ^1^Department of Biological Sciences, University of Memphis, Memphis, TN 38152, USA; ^2^Department of Computer Science, University of Memphis, Memphis, TN 38152, USA

**Background**

The detection of differentially expressed (DE) genes between two or more biological conditions is an essential step in the search for candidate disease genes, drug targets, and discriminative biomarkers. Although widely used for this task, DNA microarrays are notorious for generating noisy data. One strategy for mitigating the effects of noise is to assay many experimental replicates. However, as this approach can be costly and sometimes impossible with limited resources, analytical methods are needed which improve DE gene identification at no additional cost.

**Materials and methods**

An important source of information for differential expression analysis comes from experiments performed on microRNAs (miRNAs). While the transcriptional roles of miRNAs are well documented, principled methods for incorporating miRNA datasets with traditional gene expression assays are lacking. To this end, I developed Noisy-Or Optimization for DifferentiaL Expression analysis (NOODLE), a novel Bayesian network-based approach for integrating miRNA-mRNA interaction networks with microarray data to improve DE gene identification. Given a dataset of interest, NOODLE provides an efficient mechanism for increasing belief that an mRNA is DE if one or more interacting miRNAs are themselves DE (and vice versa).

**Results**

I first apply NOODLE to synthetic datasets, achieving more accurate DE gene identification than the popular *limma* method over a wide range of network topologies and configurations. Using two publicly available human cancer datasets, I next demonstrate how use of NOODLE increases experimental power by as much as a factor of four. Finally, I apply NOODLE to a recent dataset interrogating expression differences between human induced pluripotent and embryonic stem cells. My results uncover important biological differences between these stem cell types that would be missed using existing methods.

**Acknowledgements**

Supported in part by the Institute for Collaborative Biotechnologies through grant W911NF-10-2-0111 from the U.S. Army Research Office.

## P3 Designing and writing software for *in silico* subtractive hybridization of large eukaryotic genomes

### Deborah Burgess^1^, Stephanie Gehrlich^2^, John C Carmen^2^

#### ^1^Department of Computer Science, Northern Kentucky University, Highland Heights, KY 41099, USA; ^2^Department of Biological Sciences, Northern Kentucky University, Highland Heights, KY 41099, USA

##### **Correspondence:** John C Carmen (carmenj1@nku.edu) – Department of Biological Sciences, Northern Kentucky University, Highland Heights, KY 41099, USA

**Background**

*In silico* subtractive genome hybridization is a method used to identify unique genetic sequences in a genome of interest by deleting regions of similarity with a control genome. The program Genomic Organismal Subtractive Hybridization (GOSH) was designed and created to complete the subtractive step of *in silico* subtractive hybridization of large eukaryotic genomes using BLASTn output. A genome file, in the .fasta format, is effectively a word document containing a long string of characters. BLASTn takes two genome files and identifies areas of alignment characterized by significant sequence overlap. BLASTn can only compare two genomes. In order to identify genes found specifically in a group of organisms (i.e. the thermally dimorphic fungi), we need to compare multiple genomes or perform sequential subtractive steps.

**Materials and methods**

GOSH was designed to subtract areas of alignment from genomes using BLASTn output files. The program accesses the alignment provided by BLASTn, identifies the nucleotides making up the shared sequences in the genome, and replaces them with Ns in the genome file. Through iterative subtractions, a database containing genomic sequence shared by the thermal dimorphs *Blastomyces dermatitidis*, *Paracoccidioides brasieliensis*, and *Histoplasma capsulatum* is created which can then be aligned with the genomes of fungi which do not exhibit thermal dimorphism (e.g. *Saccharomyces cerevisiae*).

**Results**

GOSH successfully used the alignment data provided by BLASTn to modify a genome sequence file by replacing nucleotides in one genome with Ns when they aligned with another fungal genome.

**Conclusions**

Initially attempts to perform *in silico* subtractive genome hybridization using GOSH to perform iterative subtractions generated a database of sequence. However, further investigation found that the file contained multiple replicates of genomic sequence. Currently, efforts are underway to determine whether this is due to GOSH, to an inherent flaw in the design of the subtractive steps, or to the numerous repeats found in the genomes of *B. dermatitidis* and *H. capsulatum.*

## P4 Tracking the molecular evolution of Pax gene

### Nicholas Johnson, Chandrakanth Emani

#### Department of Biology, Western Kentucky University, Bowling Green, KY 42101, USA

##### **Correspondence:** Nicholas Johnson (traydreein@gmail.com) – Department of Biology, Western Kentucky University, Bowling Green, KY 42101, USA

**Background**

The purpose of this study is to trace the molecular evolution of the Paired Box (PAX) gene that was frequently expressed in diverse cancers and was crucial for growth and survival of cancer cells. The long-term goal of the study is to identify conserved domains of the PAX protein in a model organism that can be suitable drug targets for plant based anti-cancerous pharmaceutical compounds.

The paired-box (PAX) genes encode a family of nine well-characterized paired-box transcription factors, with important roles in development and disease. PAX genes are primarily expressed during embryo development, but very frequent expression was observed in diverse tumor cell lines such as lymphoma, breast, ovarian, lung, and colon cancer. A phylogenetic analysis of the PAX protein will identify crucial molecular elements that are implicated in cancer cell survival.

**Materials and methods**

In the present study the Human PAX1 protein sequence was used as a reference to retrieve homologous sequences using PSI-BLAST. A neighbor joining phylogenetic tree was developed using MEGA6.

**Results**

The conserved domains of PAX gene were identified as HTH – Helix-Turn-Helix that are shown to mediate responses to stress including exposure to heavy metals, drugs, or oxygen radicals across life forms. These could be novel targets for treatment as expression of PAX2 domain has been linked with cell survival cell migration and invasion. The generated neighbor-joining phylogenetic tree showed that the ancestral form of PAX traces back to the American alligator with another close relative in a model organism, zebrafish. PAX homologs were present extensively in birds with the most evolved form in the Golden-collared Manakin.

**Conclusions**

The bioinformatics analysis proved crucial in identifying a model organism for researching a plant based cancer treatment in the form of zebrafish, which is already being used as a model organism to study cancer. Present research in our lab already established the anti-cancerous action of plant based extracts on diverse cancers in terms of reduced cell proliferation. The study thus suggests an effective tool to research plant based treatments of diverse cancers by identifying the crucial molecular domains as targets.

## P5 Identifying genetic differences in thermally dimorphic and state specific fungi using *in silico* genomic comparison

### Stephanie Gehrlich^1^, Deborah Burgess^2^, John C Carmen^1^

#### ^1^Department of Biological Sciences, Northern Kentucky University, Highland Heights, KY 41099, USA; ^2^Department of Computer Science, Northern Kentucky University, Highland Heights, KY 41099, USA

##### **Correspondence:** John C Carmen (carmenj1@nku.edu) – Department of Biological Sciences, Northern Kentucky University, Highland Heights, KY 41099, USA

**Background**

Thermally dimorphic fungi such as *Blastomyces dermatitidis* and *Histoplasma capsulatum* have the rare, amongst fungi, ability to cause invasive infections in otherwise healthy individuals. In order to better understand what makes these fungi different from state specific fungi, we have used two bioinformatics methods to compare dimorphic and non-dimorphic fungal genomes.

**Materials and methods**

First, we compiled a list of “missing” enzymes using BLAST to search for homologs of *Saccharomyces cerevisiae* proteins in *B. dermatitidis* and *H. capsulatum*. Students searched for the components of 24 *S. cerevisiae* pathways in *H. capsulatum* and 38 *S. cerevisiae* pathways in *B. dermatitidis*.

Second, biological sciences and computer science students collaborated to create GOSH, software designed to be used to complete *in silico* subtractive genome hybridization of large fungal genomes when combined with BLASTn. A file containing thermal dimorph-shared genome sequences was created by performing multiple alignments and subtractions between the thermally dimorphic fungi of interest. This list of sequences was use to interrogate the genome the yeast *S. cerevisiae* and the genome of the mold *A. fumigatus.*

**Results**

We found 29 *S. cerevisiae* proteins from various different pathways lacking a clear homolog in *H. capsulatum*. Similarly, we found 39 missing components when we searched for *S. cerevisiae.*

Preliminary results of *in silico* subtractive genome hybridization using GOSH combined with BLASTn are promising. The iterative analysis pipeline identified multiple genome sequences found in dimorphic fungi but not one of the state-specific fungi.

**Conclusions**

We started two separate avenues of *in silico* genome comparison to identify genomic differences between thermally dimorphic fungi and state specific fungi. Both methods yielded results confirming that the dimorphic fungal genomes differ from state-specific fungi. Efforts are currently underway to expand on and characterize these differences.

## P6 Identification of conserved genomic regions and variation therein amongst *Cetartiodactyla* species using next generation sequencing

### Kalpani De Silva^1^, Michael P Heaton^2^, Theodore S Kalbfleisch^3^

#### ^1^Interdisciplinary Studies Program: Specialization in Bioinformatics, University of Louisville, Louisville, KY 40292, USA; ^2^United States Department of Agriculture, Agricultural Research Service, United States Meat Animal Research Center, Clay Center, NE 68933, USA; ^3^Department of Biochemistry and Molecular Genetics, School of Medicine, University of Louisville, Louisville, KY 40292, USA

##### **Correspondence:** Theodore S Kalbfleisch (ted.kalbfleisch@louisville.edu) – Department of Biochemistry and Molecular Genetics, School of Medicine, University of Louisville, Louisville, KY 40292, USA

**Background**

Next Generation Sequencing has created an opportunity to genetically characterize an individual both inexpensively and comprehensively. In earlier work produced in our collaboration [1], it was demonstrated that, for animals without a reference genome, their Next Generation Sequence data can be mapped to the reference genome of another animal from which it has recently evolutionarily diverged producing a wealth of data on regions which have been evolutionarily conserved, and variation therein which has been tolerated.

**Materials and methods**

Since then, 16 different animals spanning 10 different Cetartiodactyla species have been sequenced and mapped to the *Bos taurus* genome at an equivalent coverage of 10X. Here, we describe this resource which is publicly available and may be found at 10x WGS of Cetartiodactyla Species Mapped to Cattle [http://www.ars.usda.gov/Research/docs.htm?docid=25590].

**Results**

Analysis of these mappings identifies genomic regions in the respective species that are highly conserved relative to cattle. Within these conserved regions, species specific alleles selected for by evolution can be identified as well as sites that vary within the respective species. Here we present a summary of non-bovine alleles that can be measured across these species relative to the Bovine reference genome, and identify those which appear to be common to the species, and those which are likely variant within the species.

**References**

1. Kalbfleisch T, Heaton M: **Mapping whole genome shotgun sequence and variant calling in mammalian species without their reference genomes.***F1000Res.* 2014; **2**:244.

## P7 Mining physiological data to identify patients with similar medical events and phenotypes

### Teeradache Viangteeravat^1, 2^, Rahul Mudunuri^1^, Oluwaseun Ajayi^1^, Fatih Şen^1^, Eunice Y Huang^1,2^

#### ^1^Biomedical Informatics Core, Children’s Foundation Research Institute, Memphis, TN 38103, USA; ^2^Department of Pediatrics, University of Tennessee Health Science Center, Memphis, TN 38163, USA

##### **Correspondence:** Rahul Mudunuri (rmudunur@uthsc.edu) – Biomedical Informatics Core, Children’s Foundation Research Institute, Memphis, TN 38103, USA

**Background**

The volume of data that is being generated in the hospital is very large and this large volume is due to the continuous collection of sequential time series data. Clinicians are expected to examine large volumes of data along with readily available electronic medical records of patients and identify correlations between dozens to hundreds of variables based on their own clinical experience to detect significant medical events. Here, we demonstrate a data mining approach applied on physiological data to identify symbolic patterns to derive patient similar matrices that will allow clinicians to identify patients with similar events and phenotypes for the purpose of predicting patient outcomes.

**Materials and methods**

**Fig. 1 (abstract P7):**
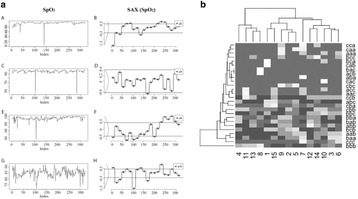
**a** Representative SAX results for SpO_2_ levels; **b** Clustering and Heatmap analysis of LRMD

We employed symbolic aggregate approximation (SAX) and piecewise aggregate approximation (PAA) techniques to convert oxygen saturation (SpO_2_) to symbolic patterns (Fig. 1a). We then applied data mining techniques called Low Rank Matrix Decomposition (LRMD) on these symbolic patterns to produce a concept vector space in which query vector and symbolic term-to-patient were projected. Resulting patients with similar events for each query are determined and compared with a control reference of 563 de-identified patients with asthma or related conditions using Precision and Recall measurements at various time intervals prior to outcomes (i.e., death, intubation, or transfer (DIT) to Intensive Care Unit).

**Results**

We found that this approach identified specific common patterns of SpO2 level among cases. For example, patterns ‘cab’, ‘bac’ and ‘acb’ exist among the patients 4, 11, 13, 15, 9, 2, 5, 7, 12, 14, 10, 3 and 6 with DIT outcomes (Fig. 1b). This results indicate their possible clinical use in the future to predict impending respiratory failure.

**Acknowledgements**

The authors would like to thank the Children’s Foundation Research Institute (CFRI), Le Bonheur Children’s Hospital, who supported this work.

## P8 Smart brief for home health monitoring

### Mohammad Mohebbi, Luaire Florian, Douglas J Jackson, John F Naber

#### Department of Electrical and Computer Engineering, University of Louisville, Louisville, KY 40292, USA

##### **Correspondence:** John F Naber (john.naber@louisville.edu) – Department of Electrical and Computer Engineering, University of Louisville, Louisville, KY 40292, USA

**Background**

A system for monitoring the moisture level and temperature of a disposable brief will be presented. The system includes a Bluetooth wireless transmitter that works in tandem with a smart phone/tablet application.

**Materials and methods**

An inexpensive disposable moisture sensor combined with a re-usable temperature sensor are used as the wireless sensors. The Bluetooth Low-Energy (BTLE) transceiver is powered by a 2032 coin cell battery having a lifetime greater than 3 years. The BTLE device collects data from the sensors and transmits the data to a smart phone/tablet. The data can be monitored locally on a smart phone/tablet or stored on a server to be analyzed remotely.

**Results**

**Fig. 2 (abstract P8):**
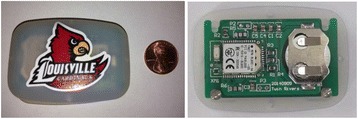
(*Left*): Top view of the hermetically sealed module with 2 brief snaps on the bottom. (*Right*): internal view showing the custom circuit board, BTLE module and coin cell holder.

A working prototype developed in the Wireless and IC design Laboratory at the University of Louisville (Fig. 2) has the following features and demonstrated characteristics:

Features:

- BTLE transmitter module sits externally on a standard disposable brief and is connected to the brief using snaps. These snaps provide electrical connection to the embedded moisture sensor.
- The BTLE equipped smart phone or tablet easily interfaces with the internet.
- The module has a low-battery alert consisting of a beep, LED or text message.
- The smart phone / tablet sends data to a server that is time stamped for statistical analysis.

Performance and Characteristics:

- The module includes a built-in temperature sensor with an accuracy of +/- 0.5 C.
- The module contains a non-replaceable coin cells that last up to 3 years with a 10 second data sampling period.
- The module has a range of at least 50’ indoors.
- The module can monitor approximate distance from the smart phone / tablet. The module costs approximately $15 in low volume.

## P9 Side-effect term matching for computational adverse drug reaction predictions

### AKM Sabbir^1^, Sally R Ellingson^2^

#### ^1^Department of Computer Science, University of Kentucky, Lexington, KY 40506, USA; ^2^Division of Biomedical Informatics, College of Medicine, University of Kentucky, Lexington, KY 40536-0093, USA

##### **Correspondence:** Sally R Ellingson (sally@kcr.uky.edu) – Division of Biomedical Informatics, College of Medicine, University of Kentucky, Lexington, KY 40536-0093, USA

**Background**

The establishment of polypharmacological networks, all the interactions between a collection of drugs and proteins, will allow for the exploration of drug re-purposing, side effect prediction, and the development of more efficacious drugs, targeting multiple proteins in a disease pathway. This project will help pave the way for the computational prediction of adverse drug reactions using a polypharmacological network built using molecular docking scores, an efficient prediction of how and how well drugs bind to a protein. The research presented here is an effort to map side-effect terms associated with a toxicity screen [1], known proteins in which drugs should not interact, to known side-effects associated with FDA-approved drugs [2] in order to test the accuracy of predictions.

**Materials and methods**

Multiple methods were tested for term matching.

1. Edit Distance determines the minimum amount of editing required to transform a source string into a destination string. Editing operations include insertion, deletion and substitution. Each operation involves a cost and a minimum cost path is found.
2. Knuth-Morris-Pratt is a pattern-matching algorithm that finds the number of times a given text pattern occurs within a text document, which has been modified to include a cost function.
3. Sense Disambiguation using meta map takes advantage of UMLS (Unified medical language system) metathesaurus, which is a large multi-purpose thesaurus containing medical and health related concepts, and builds concept networks, relating different concepts and their synonyms.
4. Language Model takes a partially formed sentence and tries to find the most appropriate word or words to form the full and proper sentence. Pubmed journal abstracts and titles were used to train the language model.

**Results**

Sense disambiguation appears to be the most accurate methods but only resolved around 75% of the terms. Multi-approach methods are being investigated to achieve the maximum number of terms matched with a high-degree of accuracy.

**Conclusion**

This significant research will help alleviate the current economic burden of developing new pharmaceuticals by innovatively utilizing massive computational power. This research will lead to the establishment of structures to use in virtual drug screenings that will predict side-effects quickly and efficiently, resulting in safer clinical trials, as fewer drugs with negative off-target effects will advance to this stage, and more affordable therapies, as drugs destined for failure will be predicted earlier in the drug discovery process. This project will address important public health concerns by providing safer and more affordable drugs.

**Acknowledgements**

This work was supported by the National Institutes of Health (NIH) National Center for Advancing Translational Science grant KL2TR000116.

**References**

1. Bowes J, Brown AJ, Hamon J, Jarolimek W, Sridhar A, Waldron G, Whitebread S: **Reducing safety-related drug attrition: the use of in vitro pharmacological profiling.***Nature rev drug discov.* 2012**; 11**(12), 909-922.

2. Kuhn M, Letunic I, Jensen LJ, Bork P: **The SIDER database of drugs and side effects**. *Nucleic acids res.* 2016; **44**(D1):D1075-D1079.

## P10 Enrichment vs robustness: A comparison of transcriptomic data clustering metrics

### Yuping Lu, Charles A Phillips, Michael A Langston

#### Department of Electrical Engineering and Computer Science, University of Tennessee, Knoxville, TN 37996-2250, USA

##### **Correspondence:** Michael A Langston (langston@tennessee.edu) – Department of Electrical Engineering and Computer Science, University of Tennessee, Knoxville, TN 37996-2250, USA

**Background**

Transcriptomic graph density and community structure remain hallmarks of putative biological fidelity. Yet these very graphs frequently have numerous maximum cliques, forcing top-down, density-based algorithms to choose a starting clique in some fashion, either randomly or by some often-arbitrary tie-breaking scheme.

**Materials and methods**

In order to help evaluate the potential effectiveness of selection strategies, we investigate the impacts of clique choice on cluster ontology enrichment and robustness. We employ yeast gene co-expression data obtained from the Gene Expression Omnibus, and create graphs in the usual fashion, by calculating all pairwise correlations and placing edges between pairs correlated at or above a selected threshold. We then run the noise-resilient paraclique algorithm to generate gene clusters.

For enrichment, we use GO p-values obtained from DAVID, and compare clusters obtained by repeatedly using the maximum clique with the highest average edge weight (correlation) to clusters obtained using the lowest average edge weight. While the use of higher weights has much intuitive appeal, we find that with proper threshold selection this choice seems to have at most a negligible effect on paraclique enrichment.

For robustness, we introduce a new metric defined as *t/(dr)*, expressed as a percentage, where *t* denotes the total number of (not necessarily distinct) gene pairs appearing together across all clusters, *d* represents the number of distinct pairs appearing together in at least one cluster, and *r* is the number of runs. Robustness thus falls between 0% and 100%. We find that paraclique scores are generally 80% or higher, demonstrating that it produces highly repeatable cluster profiles regardless of the particular starting clique chosen.

## P11 Deep neural networks for transcriptome-based cancer classification

### Rahul K Sevakula^1^, Raghuveer Thirukovalluru^1^, Nishchal K. Verma^1^, Yan Cui^2^

#### ^1^Department of Electrical Engineering, Indian Institute of Technology Kanpur, Kanpur, India 208016; ^2^Department of Microbiology, Immunology and Biochemistry, University of Tennessee Health Science Center, Memphis, TN 38163 USA

##### **Correspondence:** Yan Cui (ycui2@uthsc.edu) – Department of Microbiology, Immunology and Biochemistry, University of Tennessee Health Science Center, Memphis, TN 38163 USA

**Background**

Deep neural networks are shown to learn complex relationships in data and provide greater generalization performance in diagnostic informatics [1, 2]. Using deep learning techniques for large-scale omics data can be computationally expensive, as it involves learning of millions of network weights. This abstract focuses on designing efficient deep learning models that are sufficient to perform transcriptome-based cancer classification with minimal computational elements. This includes the use of a two layered stacked de-noising sparse auto-encoders (SDSAE) [3] to generate a feature representation that is 200 fold smaller than the input representation, and then use a novel fine-tuning method for improved classification performance.

**Materials and methods**

One of the main challenges in solving complex supervised machine learning problems is to come up with good features. SDSAE in context of Deep Learning is widely used in a variety of tasks for generation of useful feature representations. SDSAE removes redundancies while learning compact feature representations and helps the network learn important statistical regularities. The reduction in number of computational elements was achieved by drastically reducing the feature representation from input layer to 1^st^ hidden layer, and a moderate reduction from 1^st^ to 2^nd^ hidden layer. The fine-tuning method on the other hand forces data i.e. feature values in the new representation to converge towards the median of the respective class samples.

**Results**

The entire procedure was validated upon the two class Prostate Tumor data [4] containing 102 samples and 10509 features. The 2 layered neural network had 10509 nodes in input layer, 100 nodes in 1st hidden layer and 49 nodes in 2nd hidden layer. Network weights connecting the layers were initialized with denoising sparse auto-encoders and fine-tuned. Support Vector Machine (C-SVM) was used for classifying the data samples in the final feature representation. Our results show that while the network had fewer computational elements, the deep learning model with fine-tuning method gave better performance than support vector machines and random forests. Table 1 reports the four-fold cross validation AUC values (Area Under ROC Curve) of several methods.

**References**

1. Qiao C, Lin DD, Cao SL, Wang YP: **The effective diagnosis of schizophrenia by using multi-layer RBMs deep networks,***IEEE bioinformatics and biomedicine 2015 (BIBM 2015).* 2015; 603-606.

2. Lena PD, Nagata K, Baldi PF: **Deep spatio-temporal architectures and learning for protein structure prediction**, *Advances in neural information processing systems (NIPS’12) 2012*. 2012; 512-520.

3. Vincent P, Larochelle H, Lajoie L, Bengio Y, Manzagol PA: **Stacked denoising autoencoders: Learning useful representations in a deep network with a local denoising criterion**, *J mach learn res.* 2010; **11***:*3371–3408.

4. Singh D, Febbo PG, Ross K, Jackson DG, Manola J, Ladd L, Tamayo P et al. **Gene expression correlates of clinical prostate cancer behavior**, *Cancer cell*. 2002; 1(2):203-209.

**Table 1 (abstract P11):** Results on prostate tumor dataset over four-fold cross validation.

Method	AUC
Random Forest	0.9340
Linear Kernel SVM	0.9565
RBF Kernel SVM	0.8950
SDSAE + Fine Tuning + Linear kernel SVM	0.9569
SDSAE + Fine Tuning + RBF kernel SVM	0.9747

## P12 Motif discovery using K-means clustering

### Mohammed Sayed^1^, Juw Won Park^1,2^

#### ^1^Department of Computer Engineering and Computer Science, University of Louisville, Louisville, KY 40202, USA; ^2^Kentucky Biomedical Research Infrastructure Network (KBRIN) Bioinformatics Core, University of Louisville, Louisville, KY 40292, USA

##### **Correspondence:** Juw Won Park (juw.park@louisville.edu) – Kentucky Biomedical Research Infrastructure Network (KBRIN) Bioinformatics Core, University of Louisville, Louisville, KY 40292, USA

**Background**

DNA motifs are short patterns in DNA sequence and are usually associated with a biological function. The existing techniques for identifying these motifs are either computationally prohibitive or stuck at a local minimum. In this study, we propose a hybrid technique which combines both profile and word-based approaches. The proposed technique has comparable performance with classical tools such as MEME [1] and Weeder [2].

**Materials and methods**

Two types of motif features were used to discriminate motifs from background subsequences. First, relative complexity (ratio of N-mer complexity to average of background N-mers complexities) was used to capture motif structure. Second, contextual features like a position-specific scoring matrix (PSSM)-based score was used to isolate a motif from its background sequence. Unlike the expectation maximization (EM) algorithm, PSSM is computed from only one sequence. In turn, the score for each motif is constant and can be computed independent of the clustering technique.

To simplify the clustering step, features were multiplied to form one composite feature. This will reduce feature space and consequently increase the speed. K-means clustering technique was used to cluster all possible N-mers into two cluster (background and candidate). To capture over-represented candidate motifs, N-mers in candidate cluster are counted and ones with high count were determined. Finally, candidates with more than one occurrence per sequence were removed.

To evaluate the proposed technique, benchmark proposed by Sandve [3] was used. This benchmark includes 50 datasets from TRANSFAC database [4]. Different performance measures were calculated in nucleotide-level of both known and predicted sites [5]. Based on true positives (TP), true negatives (TN), false positives (FP) and false negatives (FN), the correlation coefficient was calculated:

$$ \mathrm{Correlation}\ \mathrm{C}\mathrm{oefficient}\ \left(\mathrm{C}\mathrm{C}\right) = \frac{\left(\mathrm{T}\mathrm{P}\kern0.05em *\kern0.05em \mathrm{T}\mathrm{N}-\mathrm{F}\mathrm{P}\kern0.05em *\kern0.05em \mathrm{F}\mathrm{N}\right)}{\sqrt{\left(\mathrm{T}\mathrm{P}+\mathrm{F}\mathrm{N}\right)\left(\mathrm{T}\mathrm{P}+\mathrm{F}\mathrm{P}\right)\left(\mathrm{T}\mathrm{N}+\mathrm{F}\mathrm{N}\right)\left(\mathrm{T}\mathrm{N}+\mathrm{F}\mathrm{P}\right)}} $$

**Results**

Results generated from the benchmark are presented in Table 2. These results represent the average of each performance measure over the 50 datasets. Weeder showed the highest sensitivity followed by our technique and MEME. Weeder has high sensitivity because it allows few mismatched in pattern search. On the other hand, our technique searches for the exact match. It was also interesting to see that Weeder has the highest sensitivity with the lowest specificity. In contrast, MEME has the highest specificity with the lowest sensitivity. In conclusion, our proposed technique was comparable with the existing techniques and its sensitivity can be improved with allowing mismatches in pattern search.

**Acknowledgements**

This work was supported by National Institutes of Health (NIH) grants P20GM103436 (Nigel Cooper, PI)

**References**

1. Bailey TL, Elkan C: **Fitting a mixture model by expectation maximization to discover motifs in biopolymers**. *Proc int conf intell syst mol biol.* 1994; **2**:28-36.

2. Pavesi G, Mereghetti P, Mauri G, Pesole G: **Weeder Web: discovery of transcription factor binding sites in a set of sequences from co-regulated genes**. *Nucleic acids res.* 2004; **32**(suppl 2):W199-W203.

3. Sandve GK, Abul O, Walseng V, Drabløs F: **Improved benchmarks for computational motif discovery**. *BMC bioinformatics.* 2007; **8**(1):193.

4. Wingender E, Dietze P, Karas H, Knüppel R: **TRANSFAC: a database on transcription factors and their DNA binding sites**. *Nucleic acids res.* 1996; **24**(1):238-241.

5. Tompa M, Li N, Bailey TL, Church GM, De Moor B, Eskin E, Favorov AV, Frith MC, Fu Y, Kent WJ: **Assessing computational tools for the discovery of transcription factor binding sites**. *Nat biotechnol.* 2005; **23**(1):137-144.

**Table 2 (abstract P12):** Results of the three methods: MEME, Weeder and K-means approach

	Sensitivity (SN)	Specificity (SP)	Correlation Coefficient (CC)
MEME	0.103	0.982	0.083
Weeder	0.202	0.960	0.096
K-means approach	0.176	0.971	0.090

## P13 Large scale discovery of active enhancers from nascent RNA sequencing

### Jing Wang^1,2^, Qi Liu^1,3^, Yu Shyr^1,2,4^ (yu.shyr@vanderbilt.edu)

#### ^1^Center for Quantitative Sciences, Vanderbilt University School of Medicine, Nashville, TN 37232, USA; ^2^Department of Cancer Biology, Vanderbilt University School of Medicine, Nashville, TN 37232, USA; ^3^Department of Biomedical Informatics, Vanderbilt University School of Medicine, Nashville, TN 37232, USA; ^4^Department of Biostatistics, Vanderbilt University School of Medicine, Nashville, TN 37232, USA

##### **Correspondence:** Qi Liu (qi.liu@vanderbilt.edu) – Department of Biomedical Informatics, Vanderbilt University School of Medicine, Nashville, TN 37232, USA

**Background**

Global nuclear run-on sequencing (GRO-seq) and precision nuclear run-on sequencing (PRO-seq) are techniques for mapping and quantifying transcriptionally engaged polymerase density genome-wide. They have been widely used for measuring RNA polymerase pausing and elongation, and condition-dependent transcription response. In addition, they provide a sensitive way to identify and quantify enhancer-derived RNAs (eRNAs), which is a robust indicator of enhancer activity.

**Results**

We developed a method to identify active enhancers from GRO/PRO-seq data. Applied the method to human 15 cell lines, over ten thousands of active enhancers were uncovered including 80% novel enhancers. Aligning histone modification data to the enhancer centers, we found that novel enhancers were flanked by expected histone modification markers of H3K4me1 and H3K27ac. Moreover, the signal flanking novel enhancers was as strong as that around known enhancers, indicating the reliability of novel active enhancers identified from GRO/PRO-seq data. In 12 of the 15 cell lines, the transcription abundance of enhancer-linked genes was found significantly higher than the expression of non-linked genes. In addition, the tissue specific genes were observed to locate remarkably closer to tissue specific enhancers than to universal enhancers, suggesting the regulation role of tissue specific enhancers on tissue specific expression.

**Conclusions**

The method provides efficient analysis of GRO/PRO-seq data for active enhancer identification. The large-scale discovery of active enhancers across multiple human cell lines provides valuable source for enhancer study.

## P14 Computationally characterizing genomic pipelines and benchmarking results using GATK best practices on the high performance computing cluster at the University of Kentucky

### Xiaofei Zhang^1^, Sally R Ellingson^2^

#### ^1^Department of Computer Science, University of Kentucky, Lexington, KY 40506, USA; ^2^Division of Biomedical Informatics, College of Medicine, University of Kentucky, Lexington, KY 40536-0093, USA

##### **Correspondence:** Sally R Ellingson (sally@kcr.uky.edu) – Division of Biomedical Informatics, College of Medicine, University of Kentucky, Lexington, KY 40536-0093, USA

**Background**

As genetic sequence data is now being used to make health care decisions, analysis tools needed for personalized medicine must be well tested and verified while establishing and maintaining competency in the state-of-the-art in both the technology and analysis. This study demonstrates the usefulness of high-confident call sets (validated genomic variations) in testing and optimizing bioinformatics pipelines.

**Materials and methods**

The Genome Analysis Tool Kit (GATK) [1, 2] best practices pipeline for genomic variation detection was used on two Illumina Hi-Seq genomic datasets obtained from a sample originating from NA12878, a participant in the HapMap [3, 4] project. One of the test datasets consists of four pairs of paired end data from different runs with an average depth of coverage of 14. The other consists of one pair of paired end data with an average depth of coverage of 58. Two high-confident call sets are used to detect the accuracy of the pipeline. The National Institute for Standards and Technology call set developed by the Genome in a Bottle Consortium incorporates several sequencing technologies and analysis methods [5] and the Illumina Platinum Genome call set requires concordance across multiple analysis methods and incorporates an inheritance structure.

**Results**

In this study, several types of alternatives in the entire workflow were evaluated.

1. Experimental conditions: one sequencing run with a higher depth of coverage has about 1% lower true positive rate and .1% higher positive predictive power than four runs with lower coverage each.

2. Computational architecture: threading to efficiently use a 16 CPU node gives a speed-up of almost 4.5 times that of using only one CPU; however, utilizing a 32 CPU node only gives a speed-up of 1.1 over that of a 16 CPU node.

3. Analysis tools: UnifiedGenotyper is about 7 times faster than HaplotypeCaller which only has about a 1-2% increase in true positive rates.

4. Comparison tools: GATK VariantEval, Useq vcfcomparator, and RTG vcfeval all produce similar comparison results.

**Conclusion**

A workflow that easily and reproducibly tests the accuracy and efficiency of a given method on a given computational platform is critical in order to confidently and cost-effectively utilize genomic sequencing in a clinical setting.

**Acknowledgements**

This work was supported by the National Institutes of Health (NIH) National Center for Advancing Translational Science grant KL2TR000116.

**References**

1. **GATK Best Practices**https://www.broadinstitute.org/gatk/guide/best-practices (accessed December 17, 2015).

2. McKenna A, Hanna M, Banks E, Sivachenko A, Cibulskis K, Kernytsky A, Garimella K, Altshuler D, Gabriel S, Daly M, DePristo MA: **The Genome Analysis Toolkit: a MapReduce framework for analyzing next-generation DNA sequencing data**. *Genome res.* 2010**; 20**(9), 1297-303.

3. Gibbs RA, Belmont JW, Hardenbol P, Willis TD, Yu F, Yang H, Ch'ang L-Y, Huang W, Liu B, Shen Y: **The international HapMap project**. *Nature.* 2003**; 426**(6968), 789-796.

4. The International Hapmap Consortium: **Integrating common and rare genetic variation in diverse human populations**. *Nature.* 2010**; 467**(7311), 52-58.

5. Zook JM, Chapman B, Wang J, Mittelman D, Hofmann O, Hide W, Salit M: **Integrating human sequence data sets provides a resource of benchmark SNP and indel genotype calls.***Nature biotech.* 2014; **32**:246-251.

## P15 Development of approaches enabling the identification of abnormal gene expression from RNA-Seq in personalized oncology

### Naresh Prodduturi^1†^, Gavin R Oliver^1†^, Diane Grill^1^, Jie Na^1^, Jeanette Eckel-Passow^1^, Eric W Klee^1^

#### ^1^Department of Biomedical Informatics and Statistics, Health Sciences Research, Mayo Clinic, Rochester, MN 55905, USA

##### **Correspondence:** Gavin R Oliver (oliver.gavin@mayo.edu) – Department of Biomedical Informatics and Statistics, Health Sciences Research, Mayo Clinic, Rochester, MN 55905, USA

^†^Contributed equally

**Background**

While high-throughput DNA-based clinical assays are becoming increasingly common, RNA-based approaches primarily remain a research activity. Nonetheless, the potential for clinical benefit offered by RNA-Seq, particularly in the field of personalized oncology, is substantial. RNA-Seq expression profiling of tumor samples may reinforce or deconvolute the findings of DNA-based testing. This is done by revealing the existence and magnitude of deviations from normal levels of gene expression in the tumor’s complex molecular landscape. Such evidence could ultimately be used to highlight and select targeted treatment options to improve patient care. However, multiple challenges exist to the routine implementation of these methods. One notable obstacle, particularly related to metastatic cancer, is the lack of normal tissue from the same site with which to compare RNA expression levels. Other challenges include the impact of reference sample size, reference tissue source, and the appropriate normalization and differential expression (DE) method for the N = 1 tumor sample. These challenges must be solved in order to categorize gene expression as normal or abnormal and therefore to unlock the potential of RNA-Seq profiling in the personalized oncology setting. We describe an approach taken within Mayo Clinic’s Center for Individualized Medicine (CIM) to compile normal reference expression ranges and thus evaluate DE in a N = 1 tumor sample. Utilizing data generated in house and by the The Cancer Genome Atlas (TCGA), we formulated a workflow and analytical methods that should inform and enable other researchers working in the personalized oncology field.

**Results**

We developed a bootstrap based confidence interval method to identify DE genes in a N = 1 patient tumor using different references. RLE (Relative Log Expression), 75^th^ Quantile and RPKM (reads per kilo base per million mapped reads) normalization methods has a minimal effect upon DE calling. An increase in the number of normal reference samples stabilized the number of DE calls. We observed higher gene level variance in the TCGA reference samples in comparison with in-house reference samples. DE calls using a reference from the same site were compared against mixed reference tissue samples from bladder, breast, uterine and lung tissues. While not ideal, if reference samples from the same tissue site are not available, mixed tissue reference samples can be used to recover some of the DE genes.

**Acknowledgements**

This work was supported by the Center for Individualized Medicine, Mayo Clinic.

**References**

The results shown here are in whole or part based upon data generated by the TCGA Research Network: http://cancergenome.nih.gov/.

## P16 Processing RNA-Seq data of plants infected with coffee ringspot virus

### Michael M Goodin^1^, Mark Farman^1^, Harrison Inocencio^2^, Chanyong Jang^1^, Jerzy W Jaromczyk^2^, Neil Moore^1^, Kelly Sovacool^3^

#### ^1^Department of Plant Pathology, University of Kentucky, Lexington, KY 40546, USA; ^2^Department of Computer Science, University of Kentucky, Lexington, KY 40506, USA; ^3^Department of Biology, University of Kentucky, Lexington, KY 40506, USA

##### **Correspondence:** Jerzy W Jaromczyk (jurek@cs.uky.edu) – Department of Computer Science, University of Kentucky, Lexington, KY 40506, USA

**Background**

Coffee is a widely traded agricultural commodity across the globe. The emerging coffee ringspot virus (CoRSV) reduces the quality of beans harvested and amount produced by infected plants [1]. Besides coffee, CoRSV also infects *Chenopodium quinoa* when incubated at 28° C (4° C above typical conditions for this plant) – an expanded host range which may indicate increasing risk to crops as global temperatures continue to rise [1]. Examining the differences in expression levels between infected and uninfected *C. quinoa* may shed light into the effect of CoRSV on gene expression and the effect of temperature on host susceptibility.

**Material and methods**

As an initial stage toward this goal, we developed methods for processing RNA-Seq data of virus-infected plants for which there is no reference genome. Our methods began with paired-end Illumina RNA-Seq data from three samples of *Chenopodium quinoa*: one sample infected with CoRSV and incubated at 28° C (28V), another sample uninfected and incubated at 28° C (28H), and the third uninfected and incubated at 24° C (24H). First, we analyzed the quality of the RNA-Seq data with fastqc [2], and trimmed low quality reads with Trimmomatic-0.30 [3]. We then aligned the trimmed reads to the viral RNA genome (GenBank accession numbers KF812525.1 and KF812526.1) [1] using Bowtie 2 [4]. We used HTSeq-count [5] to determine the number of reads that mapped to each viral gene. Reads from the uninfected samples which mapped to viral genes were examined for the possibility of artifacts arising from sample bleeding. To assist with this examination, we wrote a Python program to visualize the layout of reads as they were arranged on the Illumina flow cell. Finally, we are in the process of building a *de novo* transcriptome assembly of the non-viral reads using trinityrnaseq-2.1.1 [6].

**Results**

**Fig. 3 (abstract P16):**
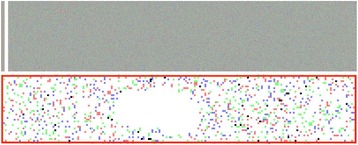
Scaled output of Tile 1301.

The results of processing the data with the Python program indicate that the three data sets were well dispersed across the flow cell. Pixels in the image are colored based on the percent composition of each data set in a particular area of the flow cell (Fig. 3). Gray areas indicate an even proportion of reads from each data set. Additionally, for each read that mapped to the viral genome from the healthy plants (28H and 24H), the identity of the nearest neighbor on the flow cell was determined. Only 3.01% of 28H reads and 2.75% of 24H reads had viral reads from the virus-infected plant as their nearest neighbors (Table 3). Thus, sample bleeding during sequencing cannot explain the vast majority of viral reads obtained from the healthy plants. Alternative hypotheses must be investigated to account for these results.

Top image: Tile 1301 at scale 100. Bottom image: red-outlined section from Tile 1301 at scale 1. Red pixels represent 28V reads, green pixels 28H reads, blue pixels 24H reads, and black pixels represent viral reads from any of the three samples.

**Acknowledgements**

This research is supported by a CAFE Seed Proposal awarded to M. Goodin.

**References**

1. Ramalho TO, Figueira AR, Sotero AJ, et al: **Characterization of Coffee ringspot virus-Lavras: a model for an emerging threat to coffee production and quality**. *Virology.* 2014; **464-465**:385-96.

2. Patel RK, Jain M: **NGS QC Toolkit: A toolkit for quality control of next generation sequencing data.***PLoS one.* 2016; **7**(2): e30619.

3. Bolger, AM, Lohse M, Usadel, B: **Trimmomatic: A flexible trimmer for Illumina sequence data.***Bioinformatics.* 2014; **30**(15)2114-2120.

4. Langmead B, Salzberg S: **Fast gapped-read alignment with Bowtie 2.***Nat methods*. 2012; **9**:357-359.

5. Anders S, Pyl PT, Huber W: **HTSeq--a Python framework to work with high-throughput sequencing data.***Bioinformatics* 2015, **31**(2):166-9.

6. Grabherr MG, Haas BJ, Yassour M, et al: **Full-length transcriptome assembly from RNA-Seq data without a reference genome.***Nat biotechnol.* 2011; **29**(7):644-52.

**Table 3 (abstract P16):** Analysis of sample bleeding plausibility.

	Total viral reads	Number of viral reads plausibly from bleeding	Fraction of viral reads plausibly from bleeding
28H reads	2,692	81	3.01 %
24H reads	2,037	56	2.75 %

## P17 Comparative transcriptomics of three *Acinetobacter baumanii* clinical isolates with different antibiotic resistance patterns

### Leon Dent^1^, Mike Izban^2^, Sammed Mandape^3^, Shruti Sakhare^3^, Siddharth Pratap^3^, Dana Marshall^2^

#### ^1^Department of Surgery, Meharry Medical College, Nashville, TN 37208, USA; ^2^Department of Pathology, Anatomy and Cell Biology, Meharry Medical College, Nashville, TN 37208, USA; ^3^Bioinformatics and Proteomics Core, Microbiology and Immunology, Meharry Medical College, Nashville, TN 37208, USA

##### **Correspondence:** Dana Marshall (dmarshall@mmc.edu) – Department of Pathology, Anatomy and Cell Biology, Meharry Medical College, Nashville, TN 37208, USA

**Background**

*Acinetobacter baumanii* is an important nosocomial pathogen in the US and worldwide. It is of great concern because it rapidly acquires antibiotic resistance (multi-drug resistant *A. baumanii* or MDRAB) and is resistant to all antibiotics, except the polymixins, in many medical centers. The time to effective antibiotic therapy correlates with patient survival, thus the initial antibiotic selection is critical. Transcriptomic biomarkers of resistance could lead to more rapid diagnosis and treatment for MDRAB infections.

**Materials and methods**

Three MDRAB clinical isolates were selected for RNAseq analysis based on varying drug resistance phenotypes (Table 4). They were cultured, in duplicate, in LB broth, and RNA was isolated using the Qiagen RNeasy kit. Illumina Genome Analyzer 2 (GAIIx) sequencing was performed using 49 bp single end reads at COFACTOR Genomics (St. Louis, MO). Assembly to reference genome (*A. baumannii* ATCC 17978) was done using CLC Genomics Workbench version 8.5.1. Transcripts mapping to genes with positive RPKM values were classified as present and a VENN overlap diagram was constructed using VENNY 2.0. Annotations were downloaded from NCBI.

**Results**

**Fig. 4 (abstract P17):**
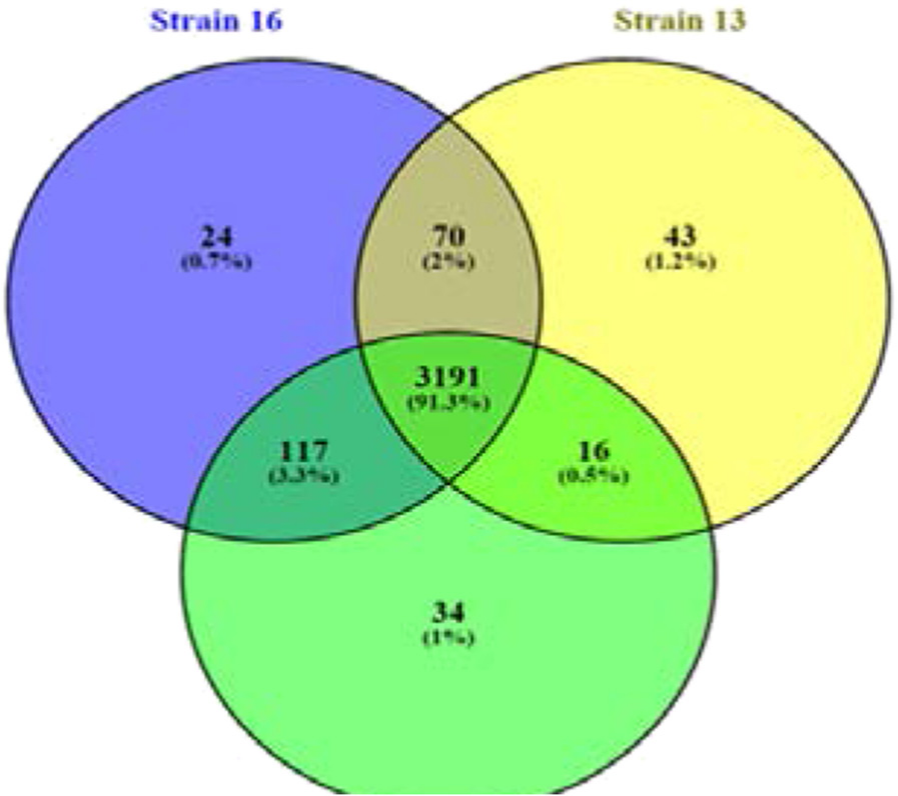
RNA-Seq Transcript overlap of three MDRAB c linical isolates shows 91% common and 0.7 – 1.2% unique transcripts among the isolates.

There were 27 – 35 million transcript read mappings to the reference genome for each of the two replicate runs per isolate. Numbers of unique and overlapping transcripts are presented in Fig. 4. Annotation of unique transcripts identified genes associated with aminoglycoside and other types of antibiotic and stress resistance, as well as virulence. As an example, only isolate 13 expresses the acetyltransferase that confers resistance to aminoglycosides, and isolate 13 is resistant to all tested aminoglycosides.

**Conclusions**

Transcriptomic analysis of three MDRAB isolates identified transcripts for genes associated with specific antibiotic resistance mechanisms. Acetyltransferase is known to confer aminoglycoside resistance in *A. baumanii,* thus its expression could be used as a biomarker in the rapid identification of aminoglycoside resistance in *A. baumanii*. These transcripts could be used as biomarkers for resistance and rapidly identified by PCR, decreasing the time required to begin effective antibiotic therapy.

**Acknowledgements**

This research was supported by The Meharry Translational Research Center (MeTRC) Grant Number U54RR026140-01 and U54 MD007593 and The Research Centers in Minority Institutes (RCMI) Grant Number G12RR003032-24S1 and G12 MD007586.

**Table 4 (abstract P17):**
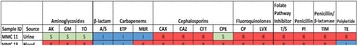
Antibiotic resistance profiles of A. baumannii clinical isolates obtained from Nashville General Hospital at Meharry. (R = Resistant, S = Susceptible, I = intermediate). Antibiotics are: AK (Amikacin), GM (Gentamycin), TO (Tobramycin), A/S (Ampicillin/Sulbactam), ETP (Ertapenem), MER (Meropenem), CAX (Ceftriaxone), CAZ (Ceftazidime), CFT (Cefitaxime), CPE (Cefepime), CP (Ciprofloxacin), LVX (Levofloxacin), T/S (Trimethoprim/Sulfamethoxazole), PI (Piperacillin), TIM (Ticaricillin/K Clavulanate), TE (Tetracycline).

## P18 Metagenomic assessment of possible microbial contamination in the equine reference genome assembly

### M Scotty DePriest^1,2^, James N MacLeod^2^, Theodore S Kalbfleisch^1^

#### ^1^Department of Biochemistry and Molecular Genetics, School of Medicine, University of Louisville, Louisville, KY 40292, USA; ^2^Maxwell H. Gluck Equine Research Center, Department of Veterinary Science, University of Kentucky, Lexington, KY 40546, USA

##### **Correspondence:** M Scotty DePriest (michael.depriest@louisville.edu) – Maxwell H. Gluck Equine Research Center, Department of Veterinary Science, University of Kentucky, Lexington, KY 40546, USA

**Background**

In a genome sequencing project, contaminating DNA from non-target organisms can result in errors in downstream analyses. These non-target organisms may include pathogens or parasites present in the original sample, as well as contaminants introduced in the sequencing process. As a genome assembly algorithm cannot distinguish between target and contaminant DNA, sequence reads from contaminants can and will be assembled into contigs. To prepare the new equine reference genome (EquCab3) for publication, contaminant contigs must be identified and screened out.

Identification of sequences as contaminants requires a metagenomic approach. Many software packages can be used to identify sequences taxonomically in metagenomics studies, but they often require very long run times and can result in many false positives. Kraken [1] addresses both of these problems by using exact matches to k-mers, rather than similarity, to identify sequences. Although Kraken was not designed specifically to identify contaminants in a eukaryotic genome assembly, it has been shown to be effective for screening the bovine genome [2]. In the current study we similarly use and test Kraken to screen EquCab3.

**Materials and methods**

To build the Kraken database, we downloaded all bacterial and viral genomes from NCBI RefSeq (11,061 genomes total) and used a k-mer length of 31. We used Kraken to search EquCab3 contigs (n = 106,319). For each flagged contig, we calculated the number of k-mer hits per kilobase of contig length. Then, we downloaded all nonredundant bacterial proteins from RefSeq to build a BLAST database (46,173,990 proteins total). We queried this database for Kraken-flagged contigs with >1 k-mer hit per kilobase using BLASTX. Contig hits with >90% sequence identity with a bacterial protein sequence along >50% of its length were considered significant. Finally, we mapped 30X Illumina HiSeq 2 × 100bp genomic DNA reads from three other horses (Thoroughbreds TB03 and TB10, Standardbred ST22) to EquCab3 using BWA MEM [3], and we calculated mapping coverage for each Kraken-flagged contig. Reads with mapping quality <10 were excluded from the calculation.

**Results**

Out of the 106,319 total EquCab3 contigs, 7,565 were identified as possible contaminants based on k-mer matches. Of the Kraken-flagged contigs, 2,257 matched more than 1 hit per kilobase, and 697 of these contained significant BLAST hits to bacterial proteins. When the short reads from three horses were mapped to these EquCab3 bacterial contigs, 217 contigs had very low (<5%) mapping coverage, indicating that the sequence was not found in the other horses. In addition, 31 bacterial contigs were mapped with >80% coverage from one or more of the test horses.

**Conclusion**

The 217 contigs in EquCab3 containing significant BLAST hits to bacterial proteins, as well as very few mapped short reads from equine genomic DNA, are likely to be contaminants. These contigs are all under 3 kb in length. Thirty-one contigs with significant BLAST hits to bacterial proteins are also well-mapped with equine short reads, suggesting that some contamination may be present in the three equine samples. Alternatively, some of these “shared contaminants” may be misidentified equine DNA, which warrants further investigation. Taxonomically, most of the contaminant sequences can be identified as commensals, including *Escherichia coli, Campylobacter jejuni*, and *Enterobacter* spp., which are known to inhabit the mammalian digestive tract. These bacteria could be introduced during sampling or any other step involving a human or horse.

Because so few Kraken-flagged contigs were confirmed as bacteria with BLAST, we conclude that Kraken alone was not sufficient to accurately identify contigs as bacteria. The screening results of metagenomics tools such as Kraken need to be further corroborated using other independent analytical methods.

**References**

1. Wood DE, Salzberg SL: **Kraken: ultrafast metagenomic sequence classification using exact alignments.***Genome biol.* 2014*;***15**:R46.

2. Merchant S, Wood DE, Salzberg SL: **Unexpected cross-species contamination in genome sequencing projects.***PeerJ. 2014;* 2: e675.

3. Li H, Durban R: **Fast and accurate short read alignment with Burrows-Wheeler Transform.***Bioinformatics*. 20029; **25**:1754–60.

## P19 Molecular evolution of cancer driver genes

### Chandrakanth Emani; Hanady Adam; Ethan Blandford; Joel Campbell; Joshua Castlen; Brittany Dixon; Ginger Gilbert; Aaron Hall; Philip Kreisle; Jessica Lasher; Bethany Oakes; Allison Speer; Maximilian Valentine

#### Department of Biology, Western Kentucky University-Owensboro, Owensboro, KY 42303, USA

##### **Correspondence:** Chandrakanth Emani (chandrakanth.emani@wku.edu) – Department of Biology, Western Kentucky University-Owensboro, Owensboro, KY 42303, USA

**Background**

The present study traces the molecular evolution of specific cancer driver genes selected from a list of 125 genes identified by the cancer genome landscapes study [1]. The purpose of the study is to identify ancestral forms of the cancer driver genes and identify the specific conserved domains during the molecular evolution in terms of gene duplications and mutational changes.

**Materials and methods**

The randomly chosen genes chosen in the specific study were ABL1, BRACA1, CASP8, DAXX, EZH2, FOXL2, GATA1, HRAS, IDH1, JAK1, MAP2K1, NOTCH1 and TP53. Protein sequences retrieved from the NCBI database by the PSI-BLAST program were subjected to multiple alignment and neighbor joining phylogenetic trees were constructed using the MEGA6 program [2].

**Results**

Comprehensive bioinformatics analysis of the resulting multiple alignments and the generated phylogenetic trees gathered valuable insights in identifying the specific molecular elements that form the basis of the specific cancer types, the related molecular processes affected across diverse life forms during the molecular evolution of the genes and suggest specific molecular targets for cancer treatment.

**References**

1. Vogelstein B, Papadopoulos N, Velculescu VE, Zhou S, Diaz LA, Kinzler KW: **Cancer genome landscapes**. *Science.* 2013; **339**:1546–1558.

2. Tamura K, Stecher G, Peterson D, Filipski A, Kumar S: **MEGA6: Molecular evolutionary genetics analysis version 6.0.***Mol biol evol.* 2013; **30**:2725-2729.

## P20 Biorepository Laboratory Information Management System

### Naga Satya V Rao Nagisetty, Rony Jose, Teeradache Viangteeravat, Robert Rooney, David Hains

#### Department of Pediatrics, The University of Tennessee Health Science Center, Memphis, TN 38103, USA

##### **Correspondence:** Robert Rooney (rrooney1@uthsc.edu) – Department of Pediatrics, The University of Tennessee Health Science Center, Memphis, TN 38103, USA

**Background**

Healthcare organizations are increasingly moving towards personalized medicine and integrating genomic information into day to day clinical decision making [1]. Biorepositories help facilitate this movement by providing the means to store, link, and analyze biological samples, clinical information, and large-scale data sets, essentially creating a platform through which clinicians and researchers in biomedical and pharmaceutical industries can identify genetic variants and mutations that cause or are associated with disease symptomology, susceptibility and/or prognosis, variation in drug and therapeutic responses, and new disease subtypes. Such information is necessary for the development and evaluation of new targeted drugs and treatment modalities, new biomarkers or more accurate diagnostic tests, proper clinical trial design, and informed life decisions by patients and their families. Effective biorepository development and operation, particularly in a hospital setting, is a complex task requiring a cohesive effort from multiple groups and technologies within the organization. At the heart of this process is a laboratory information management system (LIMS) that supports a workflow dependent upon real-time patient consenting and integration of patient data from the Electronic Medical Record (EMR), accurate and efficient sample collection, processing, storage, and distribution, and reliable integration of analysis data. The LIMS must be customizable to fit a laboratories’ equipment and procedures while still effectively protecting personal health information.

**Materials and methods**

**Fig. 5 (abstract P20):**
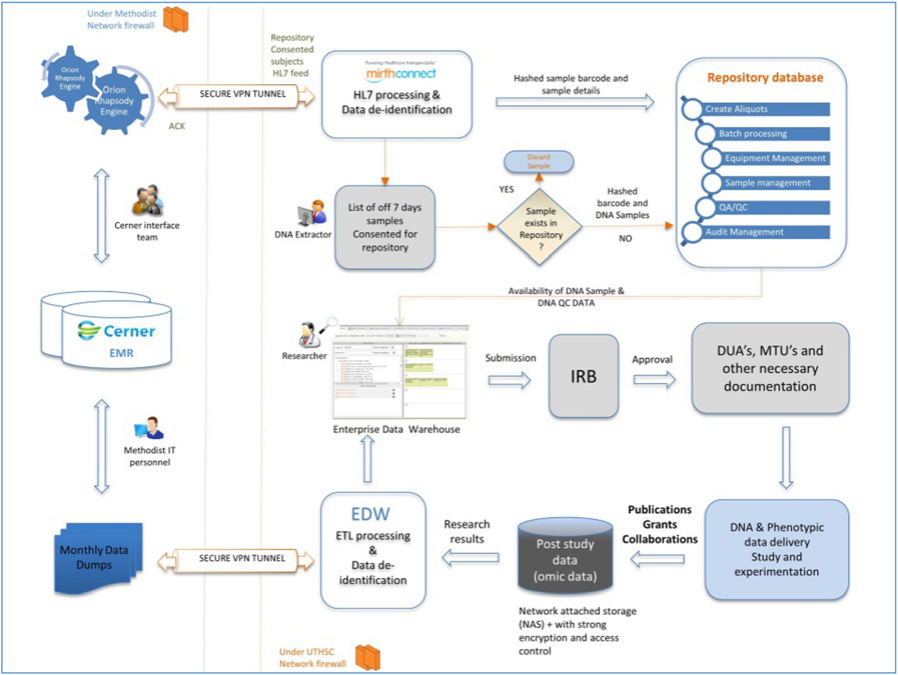
Overall infrastructure for BIG initiative.

In order to facilitate functionality tailored to biorepository needs we have built an agile and streamlined LIMS infrastructure that is cost effective, provides improved flexibility for high-throughput laboratory workflow, and has a modular design to facilitate modification and installation of new equipment and data systems (Fig. 5). The BLIMS has two major components: EMR interfaces and a template driven LIMS application. EMR interfaces were developed using open source Mirth Connect interface software using HL7 [2]. The LIMS system was developed with an interface built using PHP, JQuery & Bootstrap for flexibility and responsiveness, MVC (Model View Controller) paradigm is used to abstract various functional components of the system for extendibility, security and understandability. MySQL with PDO (PHP Data Objects) is used for data storage and manipulation. Server side functionality provides features like customizable sample templates, batch mode sample collection, and support for data import from custom laboratory equipment, multiform import in XML, CSV or TXT formats. QA/QC, tracking equipment, sample locations, statuses, and strong security with grid based access control and completed audit log management.

**References**

1. Ginsburg GS, Kuderer NM: **Comparative effectiveness research, genomics-enabled personalized medicine, and rapid learning health care: A common bond.***J clinical oncol.* 2012; **30**(34):4233-4242.

2. Viangteeravat T, Anyanwu MN, Nagisetty VR, Kuscu E, Sakauye ME, Wu D: **Clinical data integration of distributed data sources using health level seven (HL7) v3-RIM mapping.***J clin bioinform.a* 2011; **1**(1):32.

